# Influence of Prior Exercise on VO_2_ Kinetics Subsequent Exhaustive Rowing Performance

**DOI:** 10.1371/journal.pone.0084208

**Published:** 2014-01-03

**Authors:** Ana Sousa, João Ribeiro, Marisa Sousa, João Paulo Vilas-Boas, Ricardo J. Fernandes

**Affiliations:** 1 Centre of Research, Education, Innovation and Intervention in Sport, Faculty of Sport, University of Porto, Porto, Portugal; 2 Porto Biomechanics Laboratory, LABIOMEP, University of Porto, Porto, Portugal; Universidad Europea de Madrid, Spain

## Abstract

Prior exercise has the potential to enhance subsequent performance by accelerating the oxygen uptake (VO_2_) kinetics. The present study investigated the effects of two different intensities of prior exercise on pulmonary VO_2_ kinetics and exercise time during subsequent exhaustive rowing exercise. It was hypothesized that in prior heavy, but not prior moderate exercise condition, overall VO_2_ kinetics would be faster and the VO_2_ primary amplitude would be higher, leading to longer exercise time at VO_2max_. Six subjects (mean ± SD; age: 22.9±4.5 yr; height: 181.2±7.1 cm and body mass: 75.5±3.4 kg) completed square-wave transitions to 100% of VO_2max_ from three different conditions: without prior exercise, with prior moderate and heavy exercise. VO_2_ was measured using a telemetric portable gas analyser (K4b^2^, Cosmed, Rome, Italy) and the data were modelled using either mono or double exponential fittings. The use of prior moderate exercise resulted in a faster VO_2_ pulmonary kinetics response (τ_1_ = 13.41±3.96 s), an improved performance in the time to exhaustion (238.8±50.2 s) and similar blood lactate concentrations ([La^−^]) values (11.8±1.7 mmol.L^−1^) compared to the condition without prior exercise (16.0±5.56 s, 215.3±60.1 s and 10.7±1.2 mmol.L^−1^, for τ_1_, time sustained at VO_2max_ and [La^−^], respectively). Performance of prior heavy exercise, although useful in accelerating the VO_2_ pulmonary kinetics response during a subsequent time to exhaustion exercise (τ_1_ = 9.18±1.60 s), resulted in a shorter time sustained at VO_2max_ (155.5±46.0 s), while [La^−^] was similar (13.5±1.7 mmol.L^−1^) compared to the other two conditions. Although both prior moderate and heavy exercise resulted in a faster pulmonary VO_2_ kinetics response, only prior moderate exercise lead to improved rowing performance.

## Introduction

Prior exercise is traditionally accepted as indispensable before participation in a subsequent vigorous exercise. Enhancing the cardiorespiratory and neuromuscular systems, “priming exercise” has been used extensively as an intervention to investigate the limitations of pulmonary oxygen consumption (VO_2_) following the onset of a subsequent exercise bout. These limitations may be due to central (O_2_ delivery and transportation to the working muscles) or peripheral factors (from convective O_2_ transport, to its diffusion and utilization in the muscles). Measurement of pulmonary VO_2_ at the mouth is accepted to reflect muscle VO_2_ during exercise [5], thus studying the VO_2_ kinetics at the onset of exercise may provide a valid insight into the factors that regulate oxidative metabolism at the muscle [6].

Previously, the kinetics of pulmonary VO_2_ response to exercise has been studied in three different intensity domains: moderate, heavy and severe [7]. For moderate exercise (at intensities below the lactate threshold), a steady-state VO_2_ is normally reached within 2–3 min of exercise onset [8]; in the heavy domain (at exercise intensities higher than the lactate threshold but below critical power), an additional complexity (VO_2_ slow component) delays the achievement of a VO_2_ steady-state [9]. During severe intensity exercise (above critical power), VO_2_ does not achieve a steady state, but continues to increase until the point of exhaustion, as VO_2max_ is reached.

It has previously been shown that the magnitude and nature of VO_2_ responses are profoundly altered by prior exercise. The increases in bulk O_2_ delivery to the exercising muscle has dramatic effects on the response to subsequence exercise. In fact, the renewed interest on this VO_2_ kinetics area was generated by the report of Gerbino et al. [10], who demonstrated that prior heavy exercise could speed the overall VO_2_ kinetics during a second bout of heavy exercise performed 6 min after the first. Typically, studies conducted on VO_2_ kinetics have involved different prior exercise intensities [10], [11], [12], group ages [6], durations of recovery time [13], [14], body positions [4], [15], [16], baseline pulmonary VO_2_ values [17], [18], [19], [20], pedal rates [21], type of exercises [2], [22], [23], [24], [25], combinations of prior warm-up [3], [26], [27], [28] and types of subsequent bouts of exercise [29], [30]. The studies that analysed specific prior intensities have shown that the subsequent exercise performance can benefited by prior heavy exercise, as a result of an increased amplitude of the primary component and a reduced amplitude of the slow component, with no change in the primary component time constant ([11], [13], [31]). While the aforementioned alterations in VO_2_ kinetics might be expected to enhance exercise tolerance, the appropriate combination of prior exercise intensity and recovery time duration can be even more important than the prior exercise intensity *per se* [14].

We are only aware of one previous study conducted at perimaximal intensities (100%, 110% and 120% of VO_2max_) [32], that demonstrated that the time sustained at VO_2max_ was higher when prior heavy exercise was performed. Nonetheless, the effects of prior exercise have not been addressed in rowing exercise in trained athletes. Given the widespread interest in the use of prior exercise, both for training and scientific purposes, it is surprising that research focused mainly on the VO_2_ pulmonary kinetics response in cycling exercise using heavy intensity prior exercise. Thus, it is unclear whether the prior exercise regimes that are ergogenic during cycle ergometry are also ergogenic during rowing ergometry. The purpose of the present study was to examine the influence of prior moderate and heavy intensity exercise on pulmonary VO_2_ kinetics and rowing performance. On the basis of cycling data from previous studies performed at the same exercise intensity used in the present study (100% VO_2max_) [3], [32], it was hypothesized that in prior heavy, but not prior moderate exercise condition, overall VO_2_ kinetics would be faster and the VO2 primary amplitude would be higher, leading to longer exercise time at VO_2max_.

## Materials and Methods

### Ethics statement

The present study was approved by the Ethics Committee of Faculty of Sport from the University of Porto. All of the participants (or parent/guardian when subjects were under 18yrs) provided informed written consent before data collection. The procedures were performed according to the Declaration of Helsinki.

### Subjects

Six nationally ranked highly trained male subjects (mean ± SD; age: 22.9±4.5 yr, height: 181.2±7.1 cm and body mass: 75.5±3.4 kg) volunteered to participate in the current study. Subjects were familiar with the laboratory testing procedures, as they were involved in previous similar evaluations. All participants avoided strenuous exercise in the 24 hrs before each testing session, and were well hydrated and abstained from food, alcohol and caffeine intake. The protocols were conducted at the same time of the day for each subject and were separated by, at least, 24 h.

### Experimental design

Subjects visited the laboratory on four different occasions over a two week period to perform the rowing ergometer exercises (Concept II, Model D, CTS, Inc.). In their first visit, VO_2max_ and the lactate threshold were determined. During each of the subsequent visits, all subjects completed exhaustive exercise at 100% of VO_2max_ with prior moderate and heavy intensity exercises and without prior exercise. All exhaustive exercise bouts were performed at the same cadence on the rowing ergometer (ranging between 30 and 40 rpm) and encouragement was given to motivate the subjects to perform their best effort.

### Incremental exercise and exhaustive bouts

An intermittent incremental protocol of 2-min step durations, with increments of 40 W per step and 30-sec intervals between each step, until volitional exhaustion, was used to assess VO_2max_ and the corresponding minimal power that elicited VO_2max_. VO_2max_ was considered to be reached according to primary and secondary criteria [33] and the VO_2_ mean value was measured over the last 60-sec of the exercise.

**Figure 1:**
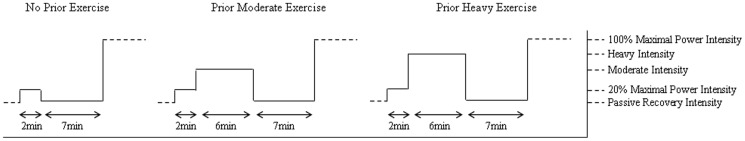
Schematic illustration of the experimental protocol. Without prior (2-min of rowing at 20% of maximal power, 7-min of passive recovery and a transition to 100% of maximal power), prior moderate (2-min of rowing at 20% of maximal power, 6-min of rowing at the moderate intensity, 7-min of passive recovery and a transition to 100% of maximal power), prior heavy (2-min of rowing at 20% of maximal power, 6-min of rowing at the heavy intensity, 7-min of passive recovery and a transition to 100% of maximal power).

A total of three experimental exhaustive conditions were investigated, conducted in randomized order. In the control condition (without prior exercise), subjects performed 2-min of rowing at 20% of maximal power (previously determined in the incremental exercise), followed by 7-min of passive recovery, and an abrupt step increment to the intensity of 100% of the minimal power that elicits VO_2max_. The subjects' then sustained their individual intensity until voluntary exhaustion. Voluntary exhaustion was defined as when the subjects' could no longer sustained the previously determined power In the other two conditions, after the initial 2-min period of rowing at 20% of corresponding minimal power that elicits VO_2max_, 6-min bouts of prior exercise were performed at moderate or heavy intensity. After the prior exercise, they had 7-min of passive recovery, which was followed by the abrupt step increment to the minimal power that elicits VO_2max_, and they maintained this for as long as possible (cf. Figure 1). VO_2peak_ and HR_peak_ were deterred as the average VO_2_ and HR values measured over the last 60-sec of the exercise in the exhaustive exercise bouts.

### Experimental measurements

VO_2_ was measured using a telemetric portable gas analyzer (K4b2, Cosmed, Rome, Italy), with the subjects breathing through a facemask with a low-dead-space. The gas analysers were calibrated before each test with gases of known concentration (16% O_2_ and 5% CO_2_) and the turbine volume transducer was calibrated by using a 3-L syringe. Heart rate (HR) was monitored and registered continuously by a Polar Vantage NV (Polar electro Oy, Kempele, Finland) that telemetrically emitted the data to the K4b^2^ portable unit. Capillary blood samples (25 µl) for determination of lactate concentrations ([La^−^]) were collected from the earlobe at 30-sec intervals immediately at the end of exercise, and during the 1^st^, 3^rd^, 5^th^ and 7^th^-min of the recovery period in the intermittent incremental protocol (Lactate Pro, Arkay, Inc, Kyoto, Japan). In the exhaustive exercise bouts, capillary blood samples were collected just before the exercise, after the prior exercise (in the 6-min of the passive recovery), and during the 1^st^, 3^rd^, 5^th^ and 7^th^-min of recovery.

### Data analysis

Firstly, occasional VO_2_ breath values were omitted from the analysis by including only those in-between VO_2_ mean ±4 standard deviation. After verification of the data, individual breath-by-breath VO_2_ responses were smoothed by using a 3-breath moving average and time-average of 5-sec [34].

VO_2_ kinetics during exhaustive exercises was assessed using 5-sec average VO_2_ data. The first 20-sec of data after the onset of exercise (cardio-dynamic phase) were not considered for model analysis with both a mono-exponential (Equation 1) or double-exponential (Equation 2) equations. For both model fits, a nonlinear least squares method was implemented in the MatLab Software to fit the VO_2_ data with each model. To allow the comparison of the VO_2_ response, data were modeled using both mono and double exponential approaches to isolate the VO_2_ fast component response. An F-Test (p>0.05) was used to evaluate whether the mono-exponential or double-exponential models provided the best fit to each data set. A T-Test (p<0.05) was employed to compare the difference between mono-exponential and double-exponential mean values. 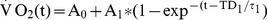
(1)

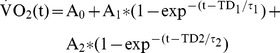
(2)where 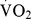 (t) represents the relative VO_2_ at the time t, A_0_ is the 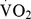 at rest (ml.kg^−1^.min^−1^) and A_1_ and A_2_ (ml.kg^−1^.min^−1^), TD_1_ and TD_2_ (s), and τ_1_ and τ_2_ (s) are the amplitudes, the corresponding time delays and time constants of the fast and slow 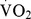 components, respectively. The mean response time (MRT) was used to represent the overall pulmonary VO_2_ kinetics response and was calculated as the sum of TD_1_ and τ_1_.

The lactate threshold was determined by visual inspection of the data as the disproportionate increase in [La^−^] as a function of work rate. In addition, to confirm the lactate threshold, it was also determined by the [La^−^]/velocity curve mathematically modelling method (least squares) [35], allowing the exact point of exponential rise in [La^−^] to be determined. Having determined the individual minimal power that elicits VO_2max_ and the lactate threshold, the work rates equivalent to 90% of the work rate at lactate threshold and to 50% of difference between the work rate at lactate threshold and at VO_2max_ were estimated and assumed to represent the moderate and heavy intensities, respectively.

### Statistics

Individual, mean and standard deviations (SD) are used for descriptive analysis for all studied variables. Measures of skewness, kurtosis and the Shapiro-Wilk test were used to assess the normality and homogeneity of the data. The differences between [La^−^] and HR mean values before and after performing the exhaustive bouts were tested using the unpaired T-Test. The differences in pulmonary VO_2_ kinetics parameters and time sustained between the exhaustive bouts preceded by moderate intensity and heavy intensity exercise and without prior exercise were tested for statistical significance using ANOVA for repeated measures. When a significant F value was achieved, the Bonferroni post hoc procedures were performed to locate the pairwise differences between the averages. Simple linear regression and Pearson's correlation coefficient were also used.All statistical procedures were conducted with SPSS 10.05 and the significance level was set at 5%.

## Results

The mean (± SD) VO_2max_ values of the subjects were 67.4±4.1 ml.kg^−1^.min^−1^, with the lactate threshold taking place at 298.3±25.6 W (corresponding to 74.9±5.7% of VO_2max_). The work rates corresponding to moderate and heavy prior exercise intensity bouts conditions were 268.5±23.1 and 348.3±16.1 W, respectively.

The basal [La^−^], baseline VO_2_ and HR mean values, just before and after the prior exercises were: 1.1±0.2 mmol.L^−1^, 6.1±1.2 ml.kg^−1^.min^−1^ and 74.6±8.4 bpm, increasing to 1.23±0.1 mmol.L^−1^, 7.3±2.2 ml.kg^−1^.min^−1^ and 83.3±7.5 bpm (p<0.05) for the without prior exercise condition, 1.14±0.3 mmol.L^−1^, 6.7±1.2 ml.kg^−1^.min^−1^ and 73.1±6.1 bpm, increasing to 2.8±0.8 mmol.L^−1^(p<0.01), 9.8±2.7 ml.kg^−1^.min^−1^ (p<0.05) and to 97.1±4.5 bpm (p<0.01), for the moderate prior exercise condition and 1.38±0.3 mmol.L^−1^, 6.3±1.5 ml.kg^−1^.min^−1^ and 73.4±4.1 bpm, increasing to 5.9±1.2 mmol.L^−1^ (p<0.01), 12.3±2.2 ml.kg^−1^.min^−1^ (p<0.01) and to 114.2±5.3 bpm (p<0.01), for the heavy prior exercise condition.

Table 1 shows the pulmonary VO_2_ kinetic parameters in the exhaustive exercise bouts, without prior exercise and with prior moderate and prior heavy exercises.

**Table 1:** Mean (± SD) values for the VO_2_ kinetics, ventilatory and metabolic parameters in the time to exhaustion bouts performed without prior exercise, with prior moderate and with prior heavy exercises.

Parameters	Without prior exercise	Prior moderate exercise	Prior heavy exercise
A_0_ (ml.kg^−1^.min^−1^)	20.48±3.49	20.91±3.48	21.61±5.28
A_1_ (ml.kg^−1^.min^−1^)	44.07±2.13	43.74±6.52	40.76±5.83
TD_1_ (s)	4.04±3.46	8.67±4.66	7.49±2.73
τ_1_ (s)	16.0±5.56 ^a^	13.41±3.96 ^b^	9.18±1.60
MRT (s)	20.05±5.44	22.08±7.46	17.24±3.29
Time sustained at VO_2max_ (s)	215.30±60.10 ^a^	238.83±50.22 ^b^	155.50±46.05
VO_2peak_ (ml.kg^−1^.min^−1^)	66.64±1.85	66.96±3.53	63.08±6.02
HR_peak_ (bpm)	179.0±15.12	182.80±9.51	182.80±14.09
[La^−^]	10.71±1.20	11.76±1.69	13.46±1.72

A_0_ = VO_2_ at rest, A_1_  =  amplitude of the fast component, TD_1_  =  time delay of the fast component, τ_1_  =  time constant of the fast component, MRT  =  mean response time (TD_1_ + τ_1_); VO_2peak_  =  peak oxygen consumption, HR_peak_  =  peak heart rate, [La^−^]  =  blood lactate concentrations. ^a^ significant different from prior moderate and prior heavy exercises; ^b^ significant different from prior heavy exercise.

**Figure 2:**
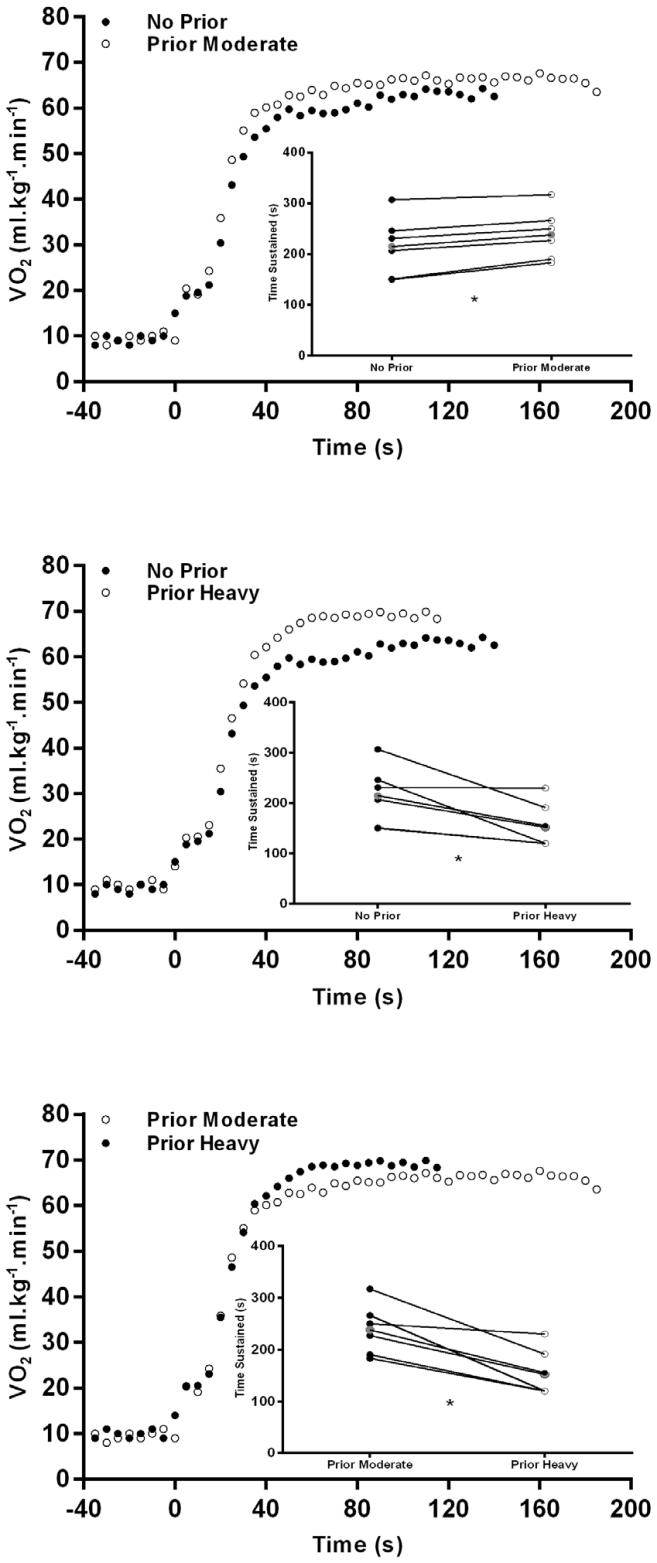
VO_2_ dynamic response of one subject performing time to exhaustion exercise bouts. After no prior exercise (closed circles) and after prior moderate exercise (open circles) (upper panel); after no prior exercise (closed circles) and after prior heavy exercise (open circles) (middle panel); after prior moderate exercise (closed circles) and after prior heavy exercise (open circles) (lower panel). The *insets* in the respective VO_2_ graphs represent the individual (full black and full white) and mean (full grey) values in the time sustained at the correspondent exercise bout released. ^*^significant differences between the two studied conditions (p<0.05).

There were no significant differences regarding A_0_, A_1_, TD_1_ and VO_2peak_ values between all three studied conditions. The overall pulmonary VO_2_ kinetics response in the fast phase was not significantly different when performing prior exercise, independently of its intensity, comparing to the condition where no previous exercise was conducted. However, τ_1_ was higher when no prior exercise was performed, comparing to the other two conditions. No significant differences were found between MRT, HR_peak_ and [La^−^]. A representative pulmonary VO_2_ kinetics and the individual and mean values of the time sustained responses at each studied condition are shown in Figure 2.

**Figure 3:**
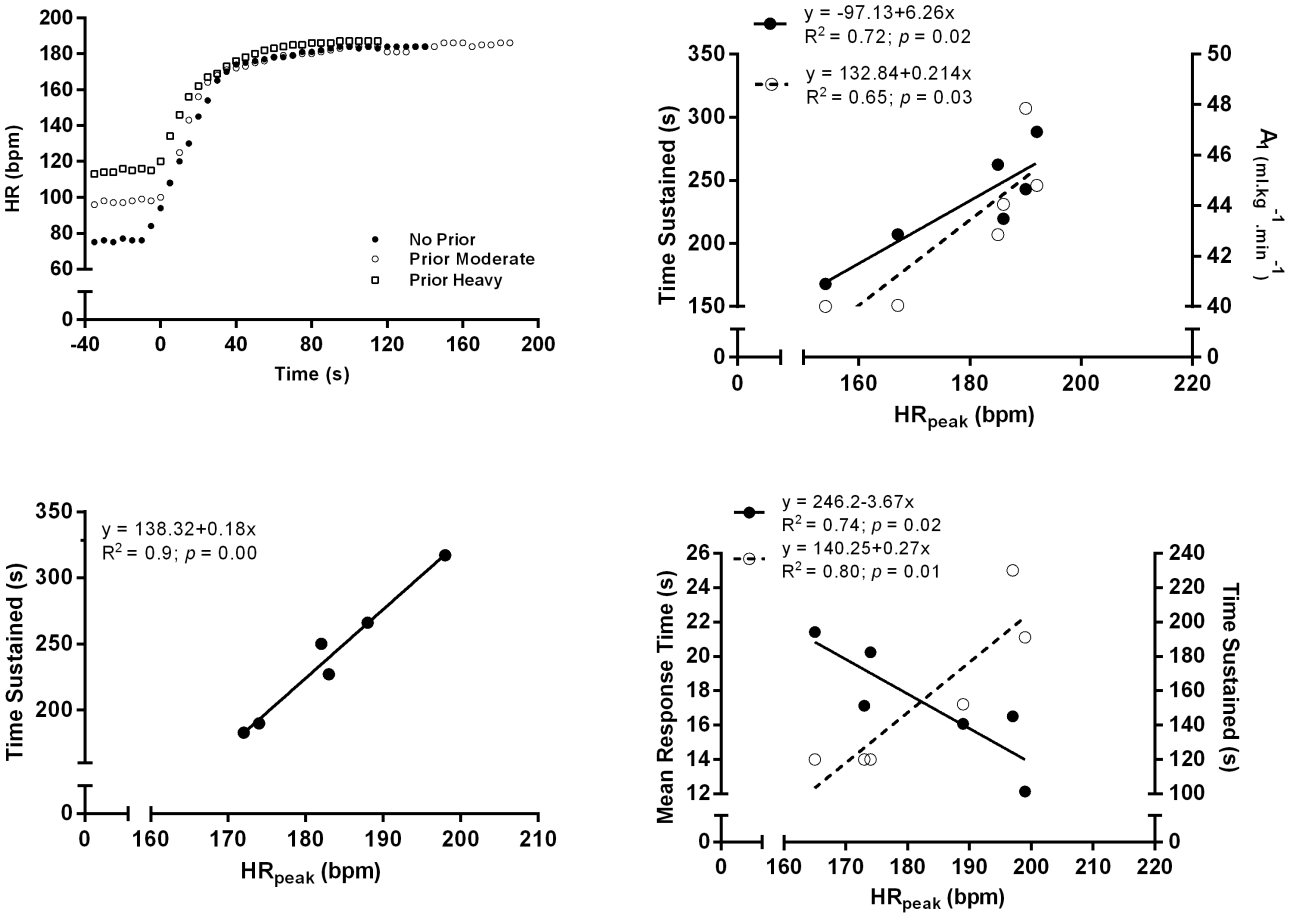
HR dynamic response of one subject performing time to exhaustion exercise bouts. After no prior exercise (closed circles), after prior moderate exercise (open circles) and after prior heavy exercise (open squares) (upper left panel); relationships between peak heart rate and time sustained (filled circles) and between peak heart rate and amplitude of the fast component (unfilled circles) when no prior exercise was performed (upper right panel), between peak heart rate and time sustained when prior moderate exercise was performed (lower left panel) and between peak heart rate and mean response time (filled circles) and between peak heart rate and time sustained (unfilled circles) when prior heavy exercise was performed (lower right panel). The regression equations, determination coefficients and significance level values are identified.

The time sustained in the exhaustive exercise bouts was longer when prior moderate exercise was performed compared to the other two studied conditions. Moreover, when a prior heavy exercise bout was implemented, the time to exhaustion was significantly shorter when compared to the without prior exercise condition. Figure 3 shows the positive relationships between HR_peak_ and the time sustained in the exhaustive bouts (all conditions). In addition, the subjects who had higher values of HR_peak_ were the ones with higher A_1_ values when no prior exercise was performed. However, in the prior heavy exercise condition, subjects who presented lower HR_peak_ values, had an enhanced fast component of VO_2_ kinetics (given by the MRT value). No significant relationships were found between VO_2peak_ and VO_2max_ and all other kinetic parameters.

## Discussion

Studies regarding the effect of “priming exercise” on VO_2_ pulmonary kinetics have been conducted mainly in cycle ergometry and using heavy intensity prior exercise domain. Only one study examined VO_2_ kinetics during rowing [36], but this study did not address the influence of “priming exercise”. The current study is the first to examine the influence of prior moderate and heavy exercises on subsequent pulmonary exhaustive rowing exercise compare to the absence of prior exercise (warm up). The main findings were that both prior moderate and heavy exercises significantly altered the pulmonary VO_2_ kinetics response to subsequent exhaustive exercise performed at 100%VO_2max_. In these two conditions, the τ_1_ was significantly shorter compared to the condition without prior exercise, in opposition to our hypothesis that prior heavy, but not moderate exercise condition, would reduce τ_1_ phase II pulmonary VO_2_ kinetics. In addition, there were significant differences among all studied conditions regarding the time sustained at VO_2max_, with higher values when prior moderate exercise was performed, again not supporting our hypothesis that time sustained at VO_2max_ would be increased when prior heavy, but not moderate, exercise would be performed.

There were significant differences in VO_2_ kinetics (τ_1_) between all studied conditions, with the values being 16.2% and 42.6% longer when no prior exercise was performed, compared to the conditions with prior moderate and heavy exercise conditions, respectively. These results for rowing are not consistent with previous studies conducted in cycling [11], [12], [13], [14], [17] or running exercise. These differences suggest that in rowing exercise, pulmonary VO_2_ steady-state is achieved faster than in cycling. It has been suggested that similarities and differences in VO_2_ kinetics between exercise sports provide insight into the physiological mechanisms responsible for the control of, and the limitations to, VO_2_ kinetics following the onset of exercise [37].

Only one study has been conducted comparing the pulmonary VO_2_ kinetic responses to step transitions to moderate and heavy intensity exercises during upright cycle and rowing ergometer exercises [38]. These authors showed that VO_2_ kinetic responses were similar between both types of exercise. This was not an expected outcome since rowing exercise engages a higher percentage of active muscular mass [39], potentially compromising muscle perfusion, particularly during heavy exercise where a larger fraction of the maximal cardiac output is used [40], Under this condition, a slower VO_2_ kinetics might be expected in rowing compared to cycling, which was not verified This outcome suggests that the greater active muscular mass engaged in rowing exercise is not, *per se*, an important explanatory factor of the differences between pulmonary VO_2_ kinetic rowing and cycling responses in moderate and heavy exercise intensities. This may also indicate that VO_2_ kinetic responses may be strongly influenced not only by metabolic constrains, but also by the muscle contraction regimen and muscle fibre recruitment profile [37]. Due to the higher intensity performed in our study (100%VO_2max_) it was expectable that bulk muscle blood flow was become even more committed compared to cycling exercise. Moreover, comparison of exercise performed with both arms and the legs reveals that muscle blood flow decreases, compared to the condition when legs or arms are exercised alone, which is explained by the sympathetic control of blood flow (muscle pressor reflex) [41]. However, this possible site of control may have been attenuated by the performance of a prior exercise and eventually resulted in a faster pulmonary VO_2_ kinetics response, which was not verified in cycling exercise. Moreover, the differences in training status of the subjects, could explain the absence of agreement between our results and the data reported in the literature, particularly for other exercise modes.

In the current study, there were differences in the time exercise was sustained at VO_2max_ between the three studied conditions, with higher values when prior moderate exercise was performed. In fact, in this condition, the time sustained at VO_2max_ was increased in 10.9% and 34.9% compared to the without prior and prior heavy exercise conditions, respectively. However, exercise time was diminished 27.8% compared to the without prior exercise condition. Recent studies have shown that exercise performance could be compromised after 6 min of cycling with a severe exercise [31], [42], enhanced after 6 min of cycling heavy exercise[31], [32] or even have no influence after 6 min of cycling at severe exercise [31]. It has been reported that prior exercise may predispose subjects to increase exercise tolerance in the subsequent bout of exercise, due to the sparing of anaerobic energy as a result of the increase in muscle aerobic energy turnover [10], [43]. This was verified in the present study by shorter τ_1_ values in the prior moderate exercise condition (compared to the without prior exercise condition), although no significant differences were found in HR kinetics between each studied condition The unexpected result that the time sustained at VO_2max_ in the prior heavy exercise condition was shorter than the other two conditions may be due to the significant higher [La^−^] values observed before the exhaustive bout was begun, compared to the prior heavy bout exercise and no prior exercise conditions.

As suggested previously, residual acidosis provides a stimulus for an increased O_2_ availability through facilitation of vasodilatation and a Bohr shift in the O_2_ dissociation curve [2], [10], [12]. However, the effects of prior heavy exercise lead also to an exaggerated accumulation of metabolites in the vascular beds in the exercised muscles and a decrease in blood pH, although muscle oxygenation was reported to be improved [2], [14]. In order to preserve the effects of prior exercise on VO_2_ kinetics and provide sufficient time for muscle homeostasis, Bailey et al. [14] reported that prior high intensity exercise can enhance the tolerance to subsequent high intensity exercise if it is coupled with adequate recovery duration (≥9 min) in between bouts. In fact, blood [La^−^], VO_2_ baseline and HR mean values were significantly elevated in the baseline period preceding the exhaustive exercise bout (5.9 mmol.L^−1^, 12.3 ml.kg^−1^.min^−1^ and 114.2 bpm), which could indicate that the recovery period may not long enough to allowed sufficient time for restoration of intramuscular high energy phosphates and/or removal of fatiguing metabolites before the beginning of the subsequent exhaustive exercise bout. The elevated [La^−^], VO_2_ baseline and HR prior to subsequent exercise could lead to a lower exercise tolerance [23], [32], [44]. This suggests that in the prior heavy exercise condition in the present study that led to the faster pulmonary VO_2_ kinetics (shown by shorter τ_1_ mean values in the prior heavy exercise condition compared to the non prior exercise one), was not the single determinant of the duration of the exercise. Instead, might do so through interaction with other physiological parameters, and, in contrast to our hypothesis, the time sustained at VO_2max_ in the prior heavy exercise condition was shorter.

In the current study, based on the positive relationship between HR_peak_ and exercise time at VO_2max,_ it was shown that the subjects who had higher HR_peak_ in all three studied conditions, were also the ones who sustained exhaustive exercises time longer. However, since no significant differences were found in HR_peak_ between all studied conditions, the O_2_ availability during exercise was similar, and so, once again, this factor is not, *per se*, the single determinant of the tolerable duration of exercise. Moreover, the subjects who presented higher A_1_ were the ones that achieved higher HR_peak_ values when no prior exercise was conducted. When prior heavy exercise was performed, negative relationships were observed between MRT and HR_peak_, as these relationships were influenced by significantly shorter τ_1_ values. In fact, a shorter τ_1_ in this condition lead to an anticipated steady-state compared to the other conditions; however, this condition has not contributed advantageous, *per si*, to a longer exercise time at VO_2max_ intensity.

Further studies to define the optimal intensity of prior exercise and subsequent recovery time required to optimise exercise performance are supported by the data from this study. However, the methodologies used to establish the intensities in both prior moderate and heavy exercise conditions (90% of anaerobic threshold and Δ50%, respectively) may have allowed that some subjects had performed them at intensities similar to important physiological boundaries: the anaerobic threshold and the critical power in the prior moderate and heavy exercise conditions, respectively. Consequently, this could have limited the rowers' performance and influenced its VO_2_ kinetics, and for that, should be construed as a possible limitation of the present study.

## Conclusions

Performance of prior moderate exercise resulted in faster VO_2_ pulmonary kinetics and also improved exercise time rowing at 100% of VO_2max_. Prior heavy exercise, although effective in accelerating VO_2_ kinetics in a subsequent exhaustive exercise, it resulted in a shorter exercise time at VO_2max_ compared to no prior exercise and prior moderate exercise. These results may have important implications for the preparation of athletes in training and competition suggesting the use of an optimal warm-up exercise intensity (and duration) with optimal recovery combination to improve performance.
